# Measuring and reducing surgical staff stress in a realistic operating room setting using EDA monitoring and smart hearing protection

**DOI:** 10.3389/fdgth.2026.1786149

**Published:** 2026-04-10

**Authors:** Merle Schlender, Ann-Christin Scherer, Tim Schneider, Jan Rennies, Verena Uslar, Dirk Weyhe, Navid Tabriz

**Affiliations:** 1 University Hospital for Visceral Surgery, Pius Hospital, Carl von Ossietzky University Oldenburg, Oldenburg, Germany; 2 Division Hearing, Speech and Audio Technology, Fraunhofer IDMT, Oldenburg, Germany

**Keywords:** cognitive workload, digital health evaluation, electrodermal activity, human factors, operating room stress, smart hearing protection, surgical simulation

## Abstract

**Background:**

Stress is a critical factor in the operating room (OR) and affects both the performance and well-being of surgical staff. Measuring and mitigating this stress can therefore improve patient safety and healthcare worker health.

**Objective:**

This study aimed to evaluate the stress levels of OR staff in a simulated surgical setting using electrodermal activity (EDA) and to assess the potential of smart hearing protection systems for stress reduction.

**Methods:**

Twenty-nine participants performed a standardized laparoscopic task in a simulated surgical setting using the LapSim® under three auditory conditions: silence, typical operating room noise (excluding alarm and speech signals), and operating room noise with simulated smart hearing protection. The smart hearing protection was simulated offline to generate an adjusted operating room soundscape rather than being implemented in real time. The smart hearing protection technology incorporates two distinct algorithms: one for speech enhancement based on blind source separation and another that reconstructs the optimal acoustic conditions for the surgical environment. Subjective workload was assessed using the Surgery Task Load Index (SURG-TLX) questionnaire, while physiological stress was measured using electrodermal activity (EDA). Cross-correlation was used to explore relationships between subjective and physiological stress measures.

**Results:**

Subjective stress levels were higher under typical operating room noise conditions (mean SURG-TLX score: 52) and markedly lower when using hearing protection with speech-filtering algorithms (mean SURG-TLX score for blind source separation: 43; mean score for the optimal condition: 42). Physiological stress, as measured by EDA, showed a reduction when smart hearing protection was used, although differences were not statistically significant. No significant correlation was found between SURG-TLX scores and EDA.

**Conclusion:**

Smart hearing protection may help reduce perceived and physiological stress during surgical procedures. The lack of correlation between subjective and physiological data highlights the importance of a multimodal approach in stress research.

## 1 Introduction

In the operating room, various stressors such as mental workload and time pressure are part of the daily routine for surgeons (1). Among these, noise is particularly burdensome for the surgical team (2), (35), as complex tasks must be performed while exposed to sound levels that often exceed 50–70 dB(A) (39). While this may not appear high in absolute terms, it exceeds recommended thresholds for healthcare environments that require sustained concentration. The World Health Organization advises that sound levels in hospital treatment and recovery rooms should be kept as low as possible, ideally not exceeding 35 dB(A), to avoid interference with rest and clinical performance (3). Although auditory attention may shift over the course of an operation and conversations can sometimes be perceived as either disturbing or even helpful (35), excessive noise levels generally contribute to reduced work quality (2). This, in turn, is associated with increased complication rates and decreased patient safety (4, 36–41). It is also important to recognize that persistent stress in surgeons may result in adverse health outcomes (2), including psychological problems (4–6). To mitigate the long-term effects of stress in clinical practice, it is essential to establish a standardized measurement approach that provides insight into stress responses in relation to various influencing factors. Developing targeted stress reduction strategies requires this kind of information.

Previous research has already addressed potential stressors in the operating room. For instance, Rieger et al. (7) demonstrated that the duration of a surgical procedure has a significant impact on perceived workload. To prevent chronic overload and associated health risks, they recommend integrating intraoperative breaks into surgical workflows. Moreover, their findings indicate that acting in the role of the primary surgeon is not associated with higher levels of subjectively perceived workload or stronger objectively measurable cardiopulmonary responses (7). In contrast, the impact of acoustic stressors has not yet been investigated in the operative setting.

Currently, stress in surgical staff is primarily assessed using subjective methods, such as the surgery task load index (SURG-TLX) (8) and the NASA task load index (NASA-TLX) (9), both of which have been applied in surgical contexts (10–13). However, there are also objective methods available to assess stress, though they have yet to be widely implemented in operative environments. These include various physiological parameters such as skin temperature, electroencephalography (EEG), electromyography (EMG), respiration, electrodermal activity (EDA), and heart rate, which remains a classical indicator of acute stress levels (14). Nevertheless, because both psychological and physical stress typically result in increased heart rate, pulse measurements alone do not allow for a clear distinction between physical exertion and emotional stress (15, 16). In contrast, electrodermal activity provides more specific insight into psychological states such as stress (17). EDA measures sweat gland activity on the surface of the skin as well as skin conductance level (SCL), expressed in Siemens, the SI unit (18).

Standard EDA measurement involves the palmar surfaces of the hands, as these areas show strong electrodermal responses (18, 19). This is attributed to the high density of sweat glands found on the palms and soles of the feet, which allows for stronger and more informative signals (20). These particular sweat glands are primarily activated by emotional arousal, which makes EDA a valuable indicator of psychological states (18). During standard palmar EDA recording, electrodes are placed on the index and middle fingers, with several placement variations including the distal phalanges, the volar surfaces of the middle phalanges, or the thenar and hypothenar regions (19). However, attaching electrodes to the hands is not feasible during surgery, as this would interfere with the performance of surgical tasks and potentially compromise patient safety. Therefore, alternative recording sites for EDA must be identified and validated.

Some studies have explored EDA measurement at non-palmar sites (21). As early as 1967, Edelberg reported on alternative locations including the shoulder, neck, and chest (20). More recently, Payne et al. (22) demonstrated that toe-based EDA recordings may yield results comparable to those obtained from fingers (22). Although Boucsein (18) investigated the wrists as an alternative site, this location proved unsuitable due to lower skin conductance and the fact that wrist sweat glands primarily serve thermoregulatory rather than emotional functions (18). A recent study further suggested that the toes and the shoulder-neck region may offer promising alternatives. These locations showed strong correlations with standard finger-based EDA measurements. Additionally, while electrodes on the toes may be perceived as uncomfortable or disruptive during surgical procedures, electrodes positioned on the shoulder-neck region are generally considered less intrusive (23). Despite these findings, no consensus has yet been established regarding the suitability of alternative EDA measurement sites.

The aim of the present study is to investigate and validate the shoulder-neck region as a practical and reliable site for EDA monitoring. This area is particularly well suited for use during surgical procedures, as it does not interfere with hand movements or clinical tasks. In this study, EDA data from the shoulder-neck area are collected alongside data from the standard palmar sites to allow for direct comparison. To further strengthen the validity of the stress assessment, heart rate data are also collected using ECG as an additional objective parameter. Furthermore, the study evaluates the use of different sound enhancement algorithms designed to suppress irrelevant auditory stimuli while preserving critical speech signals for the wearer. The goal is to examine whether targeted noise reduction helps lower perceived stress in the operating room, enhances focus, and improves task performance.

More specifically, this study pursues two main objectives: Aim (1) is to validate the shoulder-neck region as a practical and reliable alternative site for EDA measurement by comparing it with standard palmar recordings. Aim (2) is to evaluate the potential of smart hearing protection systems, including different sound enhancement algorithms, to reduce perceived and physiological stress in a simulated surgical environment.

This study has the potential to support the development of reliable methods for objective stress assessment in medical professionals and other occupational groups in which palmar EDA measurement is not feasible. Additionally, it may provide insight into whether sound enhancement technologies, already used in other industries to reduce mental workload, could be effectively applied in surgical settings. Ultimately, the findings may contribute to identifying stressors, optimizing workflows, and protecting te mental health and well-being of clinical personnel.

## 2 Materials and methods

### 2.1 Participants

A total of 29 individuals participated in the experiment (20 female, 9 male). The age of the participants ranged from 20 to 50 years (average age: 25.4 years). All participants had no medical training or professional surgical experience. Participants were recruited via online advertisements, and the data collection period spanned six weeks. At the outset, all participants completed a questionnaire to assess their current mental workload. This provided a baseline measure of their mental state prior to the study tasks, independent of the experimental procedures. The study was approved by the University of Oldenburg local ethical committee under the local file no. 2024-111 on August 8, 2024.

### 2.2 Measurement setup

For the purpose of biosignal acquisition in this study, devices from Plux Biosignals (Explorer Kit; Plux Biosignals, Lisbon, Portugal) were employed. The biosignalsplux 4-channel hub enables the recording of raw physiological data across four independent channels and transmits these data wirelessly via Bluetooth to a receiving device. Due to its compact and portable design, the system is well suited for use in mobile and dynamic settings (24). Two types of sensors were utilized: one for EDA and one for ECG. The EDA sensor captures dynamic changes in the electrical conductance of the skin, which are modulated by sympathetic nervous system activity, such as changes in sweat gland function and perspiration levels (25). The ECG sensor employs a low-noise differential triode configuration, optimized for local signal acquisition and minimal signal distortion (26). Both EDA and ECG data were recorded at a sampling rate of 500 Hz.

**Figure 1:**
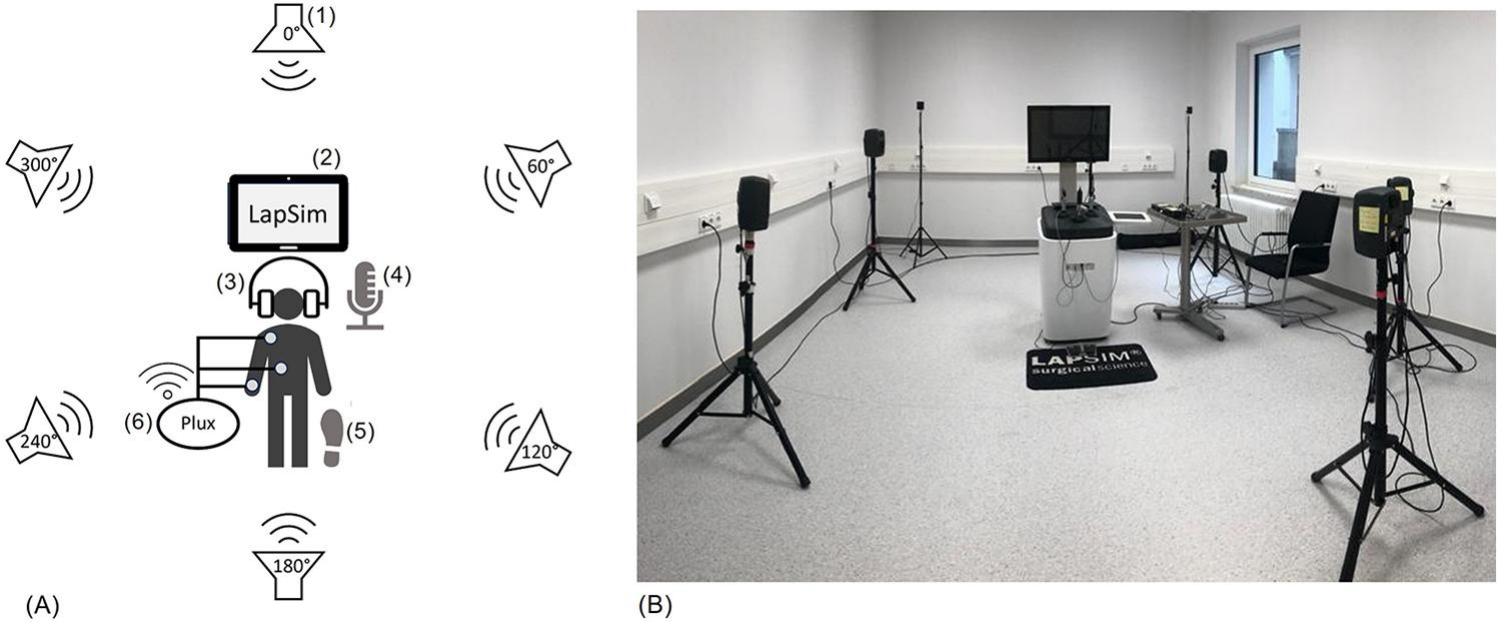
**(A)** schematic representation of the experimental setup: 1) speaker system (360° array), 2) laparoscopic simulator (LabSim), 3) hearable device with sound enhancement, 4) microphone, 5) foot pedal, 6) plux system (biosignal acquisition); **(B)** laboratory setup of the experiment.

In addition, a virtual reality-based laparoscopy simulator (LapSim, Surgical Science, Gothenburg, Sweden) was used as part of the experimental setup. The LapSim system incorporates haptic feedback to simulate surgical scenarios and has been validated in previous research, including the study by Woodrum et al. (27). To create a realistic acoustic environment, a six-speaker array was employed to play authentic audio recordings from real surgical settings. The background noise consisted of ventilation sounds, movements of personnel within the room, and various medical machines, with the noise level at the participants’ location calibrated to 70 dB sound pressure level (SPL), which is consistent with values reported for real operating rooms. Raw biosignal data were processed using OpenSignals software (Public Build 2022-05-16; Plux, Lisbon, Portugal), which supports real-time monitoring and visualization of multisensor data streams (24). Data preprocessing and visualization were conducted using MATLAB (Version R2024b, MathWorks, Natick, Massachusetts), while statistical analyses were carried out with IBM SPSS Statistics [Version 28.0.1.0(142), IBM, New York, USA]. An overview of the complete experimental setup is provided in Figure 1.

### 2.3 Experimental procedure

All participants provided written informed consent prior to their inclusion in the study. As part of this process, they were informed in detail about the experimental procedures and the handling of the collected data. In addition to general demographic information, participants' handedness was recorded, as it was essential for the correct placement of the electrodes. Following the placement of the electrodes for EDA and ECG signal acquisition, the system was connected via cables and paired via Bluetooth with a laptop. Additionally, a hearing screening was conducted to ensure that all participants had normal hearing (pure tone thresholds not exceeding 20 dB hearing level, REFs).

Subsequently, a five-minute baseline measurement was performed during which participants remained motionless. The main experimental phase consisted of six different conditions, incorporating both single-task and multi-task scenarios, all conducted within a simulated operating room sound environment.

In the single-task conditions, participants completed a highly realistic laparoscopic simulation task involving the placement of needles into a retrieval bag using laparoscopic tools (LapSim task “lifting and grasping”; approximately 3–4 min). The multi-task conditions combined this simulation with two additional tasks: a speech comprehension task based on the CCOLSA (concurrent Oldenburger Satztest) paradigm (28), in which participants were required to repeat the speech of designated target voices. Because target voices changed over the course of the task, participants also had to continue monitoring non-target voices while focusing on the current target, thereby imposing high demands on auditory attention. The second additional task was an alarm detection task in which participants were instructed to respond only to target alarms using a foot switch while ignoring all non-target signals. The target signals in the speech comprehension task had a signal-to-noise ratio of +3 dB relative to the background noise, while the alarm signals had a signal-to-noise ratio of +10 dB.

Across the various conditions, participants wore headphones simulating smart hearing protection technology for sound enhancement. Three different modes of noise mitigation were implemented were implemented: (1) no signal processing, mimicking a passive damping scenario in which all sounds are attenuated (referred to as damped in the following), (2) speech enhancement technology implemented as simulated blind source separation (BSS) technology which attenuates all non-speech sounds by 10 dB, and leaves all speech sounds unmodified, effectively increase the signal-to-noise ratio by 10 dB (referred to as BSS in the following). Because a BSS technology enhances all speech sounds (including potentially irrelevant and disturbing speech) and attenuates all non-speech sound (including potentially relevant alarm signals), an (3) even more targeted sound enhancement was implemented. This was achieved by directly reproducing the current target voices and the target alarm signals at the listener's ear. This corresponds to a scenario in which all sound sources are digitally connected to the listener and a signal routing system is implemented such that all relevant sounds (voices and alarms) reach the listener in an amplified way while all other sounds (irrelevant speech and alarms) are attenuated (referred to as direct in the following). The order of conditions was randomized and balanced across participants to control for sequence effects. An overview of all six conditions, including the associated tasks and applied audio algorithms, is provided in Table 1.

**Table 1:** Overview of hearable configurations and task requirements across experimental conditions.

Condition	Hearable configuration	Laparoscopy simulation	Speech comprehension	Alarm reaction
single task (st)	no hearable	✓	✗	✗
single task_damped_ (st_d_)	switched off	✓	✗	✗
multi task (mt)	no hearable	✓	✓	✓
multi task_damped_ (mt_d_)	switched off	✓	✓	✓
multi task_BSS_ (mt_BSS_)	simulated BSS	✓	✓	✓
multi task_direct_ (mt_direct_)	Direct transmission of relevant target signals	✓	✓	✓

Prior to the experimental phase, participants completed a structured training session consisting of six individual trials to practice each task in isolation as well as in combination, ensuring a thorough understanding of all experimental procedures. After each condition, participants completed the SURG-TLX questionnaire, a widely used instrument for assessing perceived workload across various domains. The SURG-TLX is designed to evaluate the cognitive and physical demands encountered by individuals interacting with human-machine systems (10). In addition, participants answered follow-up questions regarding perceived task difficulty and their focus of attention during each condition. Upon completion of the final condition, all sensors and electrodes were removed.

### 2.4 Statistical analysis

Data from all participants who completed all six trials in full were included in the analysis.

The results from the baseline EDA measurement, as well as the EDA and ECG data from the individual experimental conditions, were analyzed separately. ECG data were processed using MATLAB's Signal Processing Toolbox to detect peak values, which were subsequently used to calculate heart rate. To ensure accurate peak detection, an individualized threshold was defined for each participant. Peaks falling below this threshold were excluded from further analysis. To prevent multiple detections within a single cardiac cycle and to reduce detection errors, a minimum interval of 60 samples between consecutive peaks was enforced. For the analysis of EDA data, a repeated-measures ANOVA was conducted with the factors recording site (finger, shoulder) and condition (st, st_d_, mt, mt_d_, mt_BSS_, mt_direct_). The recorded signals were normalized, and cross-correlations between the signals from the two recording sites were computed. To quantitatively evaluate the correlation coefficients and to infer the effect of the individual conditions, an additional repeated-measures ANOVA (factor: condition) was performed using the maximum value of each calculated cross-correlation. Since the assumption of sphericity was violated in some comparisons, the Greenhouse-Geisser correction was applied throughout the entire analysis.

## 3 Results

### 3.1 EDA

To examine the results of the EDA data, both the resting measurement and the measurements taken during the task conditions were analyzed. At rest, the mean SCL value measured at the fingers was 7.87 ± 2.25 μS, while the value recorded at the shoulder was 5.25 ± 2.92 μS. To compare SCL values across different recording sites during the main task, an ANOVA was conducted using the average skin conductance levels from each site across all six conditions, including the additional resting condition.

Table 2 presents the means and standard deviations of the recording sites across all conditions.

**Table 2:** Means and standard deviations of skin conductance levels for finger and shoulder measurements across all six conditions.

Condition	SCL finger/μS	SCL shoulder/μS
single task (st)	7.53 ± 2.72	5.31 ± 3.16
single task_damped_ (st_d_)	7.47 ± 2.45	5.35 ± 3.16
multi task* (mt)	7.75 ± 2.64	5.56 ± 3.14
multi task_damped_ (mt_d_)	7.71 ± 2.64	5.57 ± 3.09
multi task_BSS_ (mt_BSS_)	7.61 ± 2.65	5.44 ± 3.05
multi task_direct_ (mt_direct_)	7.66 ± 2.75	5.53 ± 3.02

* This condition serves as the reference, i.e., it corresponds to standard clinical practice.

**Figure 2:**
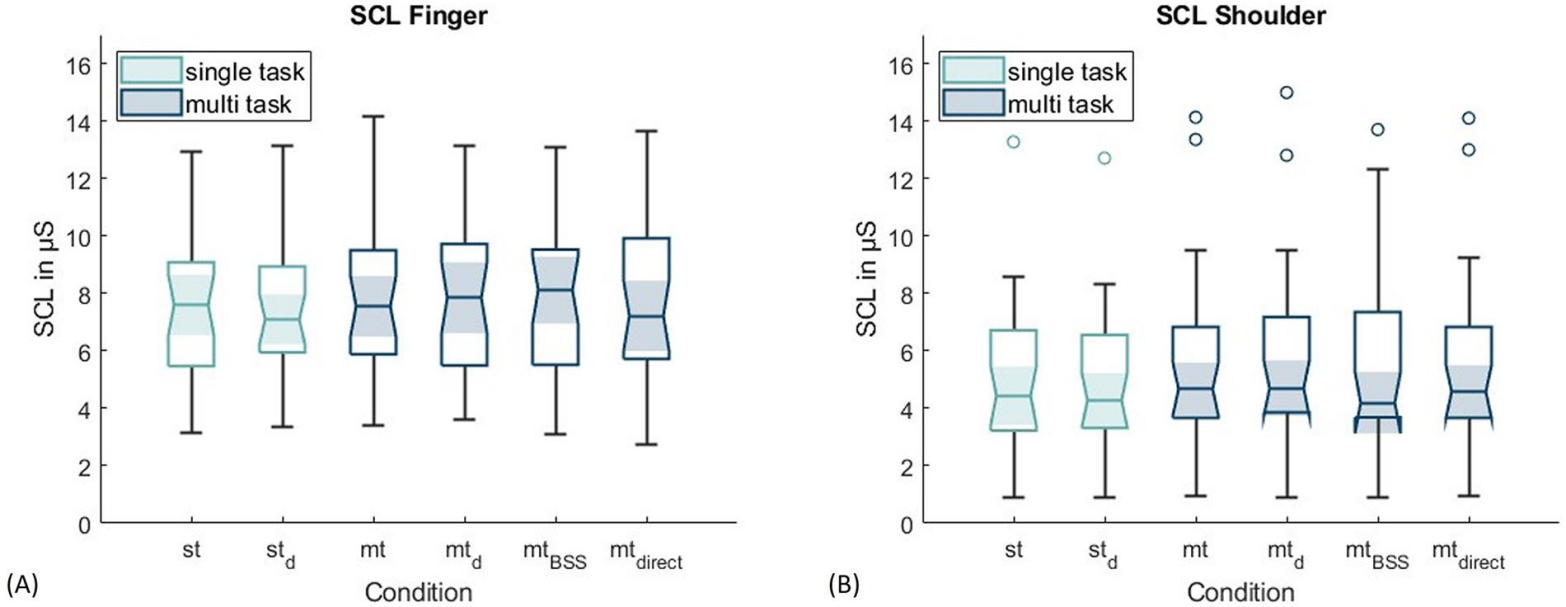
Boxplots of mean SCL values across all experimental conditions, measured at **(A)** the finger and **(B)** the shoulder.

In addition, Figure 2 presents boxplots of the participants’ mean SCL values across all conditions for the finger and shoulder recording sites.

The figure illustrates that, overall, mean SCL values are higher at the fingers compared to the shoulder. Across all conditions, the mean SCL value at the fingers was 7.62 ± 2.61 μS, while at the shoulder it was 5.48 ± 3.06 μS. An ANOVA comparing SCL values at both recording sites, including the resting condition, revealed a significant effect, F(1, 28) = 8.445, *p* = .007, partial *η*^2^ = .232, confirming that SCL values at the shoulder are significantly lower than those at the fingers. When examining individual conditions, no substantial differences were observed. This impression was confirmed by the ANOVA, which showed that the different conditions did not have a significant effect on participants’ SCL values, F(2.037, 57.043) = 0.514, *p* = .604, partial *η*^2^ = .018. Likewise, no significant interaction effect between recording site and condition was found, F(1.423, 39.844) = 0.473, *p* = .562, partial *η*^2^ = .017.

To examine the relationship of EDA between the different electrode sites, a cross-correlation analysis was conducted. The resulting means and standard deviations of the correlation coefficients for the various conditions are summarized in Table 3 for clarity. For the resting condition, the correlation coefficients were found to be 0.996 ± 0.006.

**Table 3:** Overview of the means and standard deviations of the correlation coefficients of EDA between finger and shoulder measurements across all conditions.

Condition	Correlation coefficient
single task (st)	0.997 ± 0.003
single task_damped_ (st_d_)	0.996 ± 0.005
multi task* (mt)	0.995 ± 0.007
multi task_damped_ (mt_d_)	0.994 ± 0.008
multi task_BSS_ (mt_BSS_)	0.995 ± 0.011
multi task_direct_ (mt_direct_)	0.995 ± 0.007

* This condition serves as the reference, i.e., it corresponds to standard clinical practice.

To enable statistical analysis of the correlation coefficients, the coefficients obtained for the six conditions were compared with one another.

**Figure 3:**
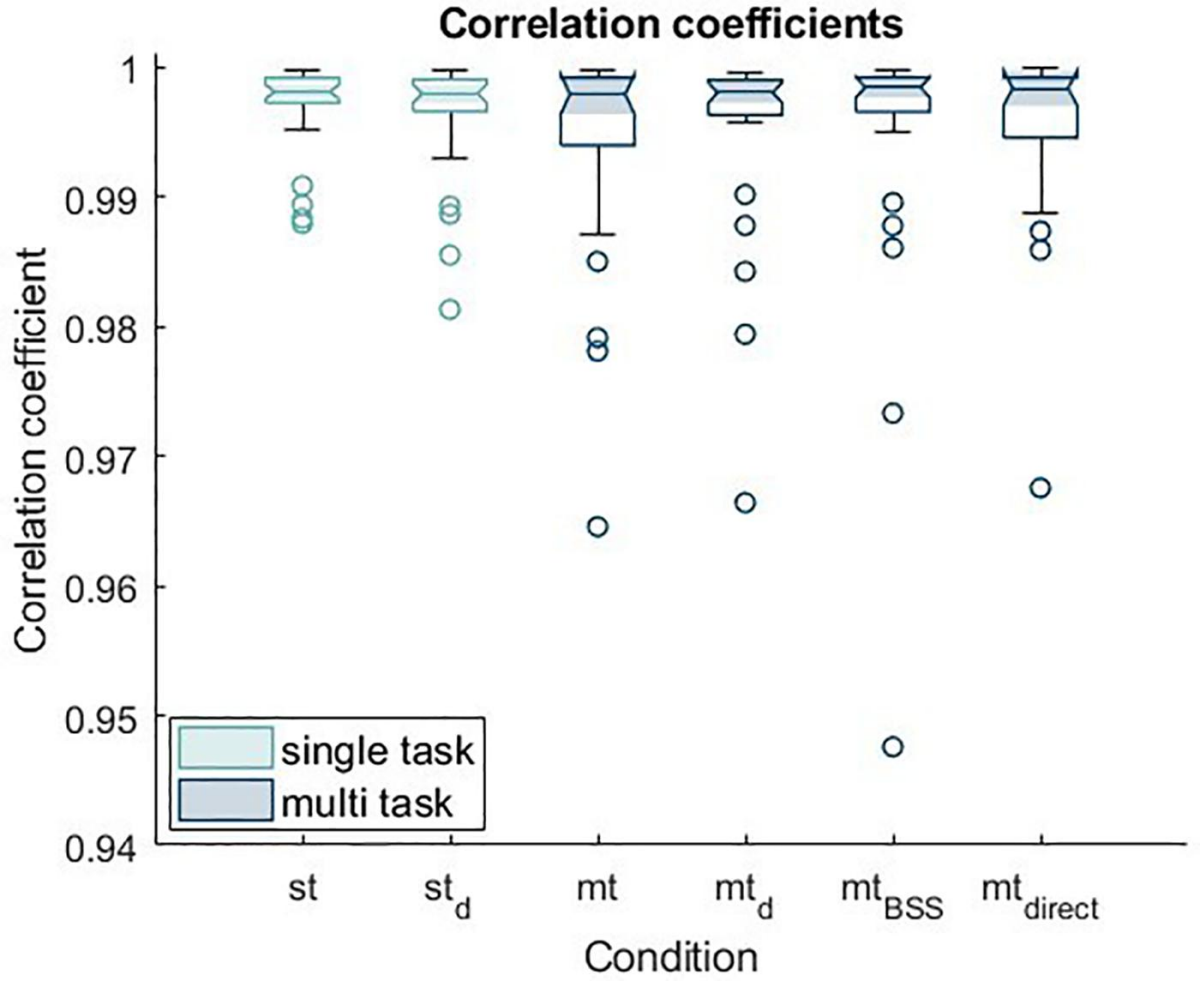
Boxplots of the mean correlation coefficients of EDA signals from the finger and shoulder across all conditions. The high correlations across all conditions indicate stable and consistent EDA signal capture at the shoulder relative to the standard finger site.

Figure 3 displays the cross-correlation coefficients for the different conditions at the finger and shoulder recording sites.

A comparison of the EDA measurements at the fingers and the shoulder reveals that the mean correlation coefficients consistently exceed 0.99 and exhibit low variability. An ANOVA conducted on the main task data also showed no significant effect of condition on the correlation coefficients, F(2.858, 80.023) = 1.501, *p* = .222, partial *η*^2^ = .051. These findings indicate that the correlation coefficients remain stable across all conditions.

### 3.2 ECG

Table 4 presents the means and standard deviations of heart rate across the different conditions as well as during the resting state.

**Table 4:** Overview of mean values and standard deviations of heart rate across all conditions.

Condition	Heart rate/BPM
Rest	74.1 ± 12.6
single task (st)	86.7 ± 14.1
single task_damped_ (st_d_)	84.8 ± 10.6
multi task* (mt)	84.0 ± 8.9
multi task_damped_ (mt_d_)	84.8 ± 9.9
multi task_BSS_ (mt_BSS_)	83.9 ± 9.8
multi task_direct_ (mt_direct_)	85.8 ± 11.4

* This condition serves as the reference, i.e., it corresponds to standard clinical practice.

The average heart rate during mental exertion was consistently higher than during the resting condition. Across task conditions, mean heart rates were relatively similar, with only small variations between single-task and multi-task settings.

**Figure 4:**
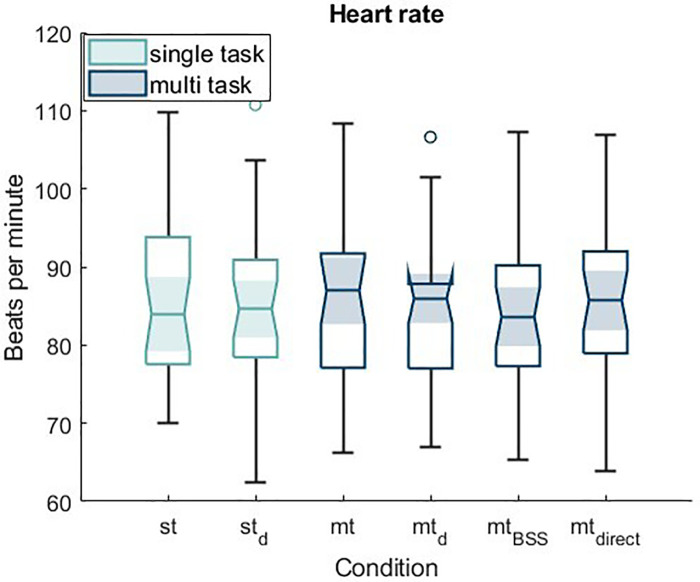
Boxplots of participants’ mean heart rate across all conditions.

Figure 4 displays boxplots of the mean heart rate across the different conditions. To allow for statistical interpretation, an ANOVA was conducted to examine whether the conditions, including the resting measurement, had an effect on heart rate (beats per minute). The ANOVA revealed a significant effect of condition on heart rate, F(3.062, 85.741) = 11.492, *p* < .001, partial *η*^2^ = .291.

Post hoc tests revealed that significant differences occurred exclusively in comparison to the resting condition (rest vs. st (single task): *p* = .004, rest vs. st_d_ (single task damped): *p* = .002, rest vs. mt (multi task): *p* = .003, rest vs. mt_d_ (multi task damped): *p* = .001, rest vs. mt_BSS_ (multi task, simulated blind source separation): *p* = .005, rest vs. mt_direct_ (multi task, direct transmission of relevant target signals): *p* < .001).

### 3.3 Subjective stress load

**Figure 5:**
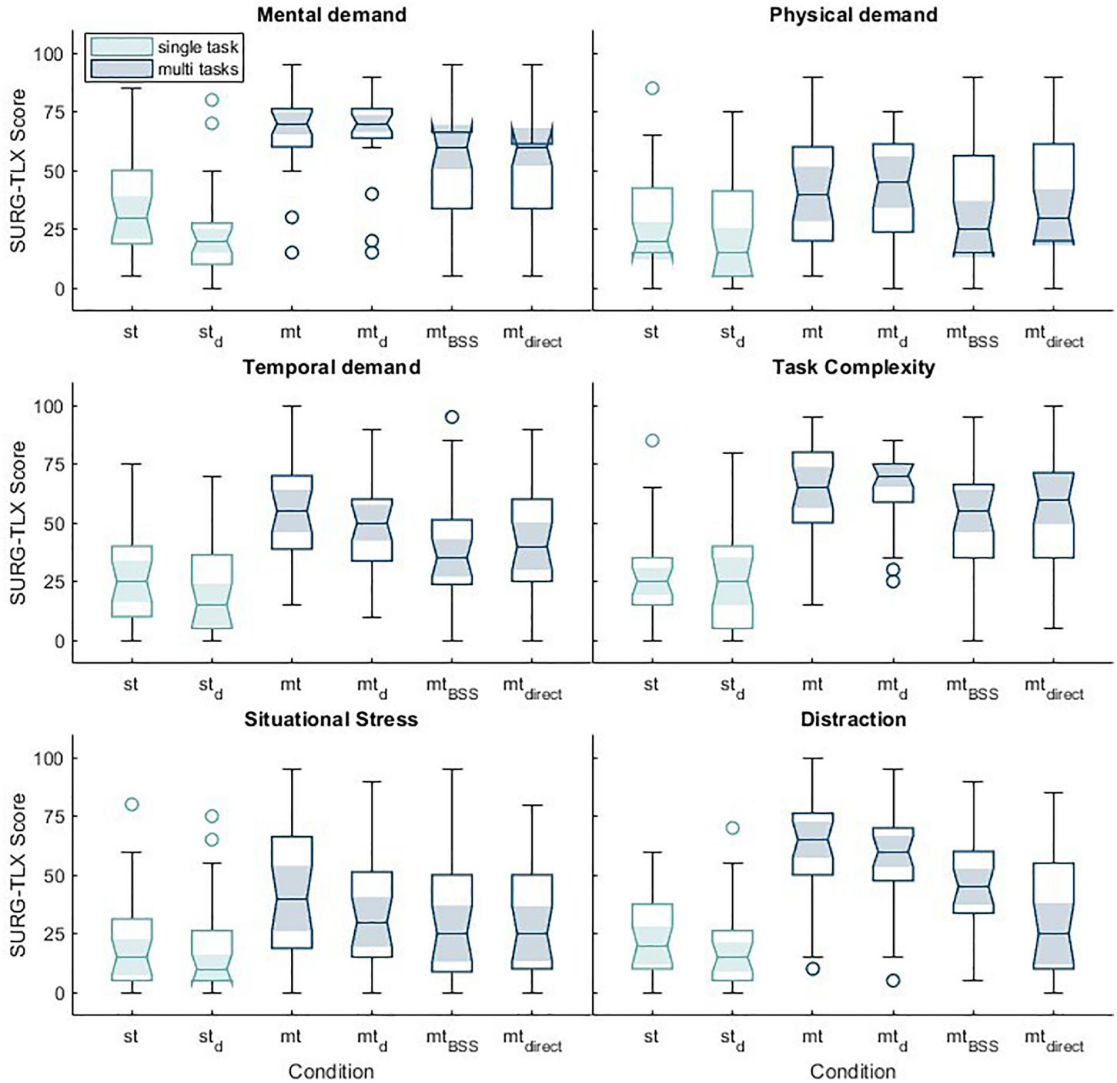
Boxplots of mean SURG-TLX scores across all conditions for each domain of the questionnaire: mental demand, physical demand, temporal demand, task complexity, situational stress, and distraction. The light blue boxplots represent the single-task conditions, while the dark-colored boxplots correspond to the multi-task conditions. Multi-task conditions show consistently higher subjective workload across all domains, with the largest differences observed in mental demand, temporal demand, and distraction.

To assess subjective stress load, the multidimensional SURG-TLX) was employed. The workload domains: mental demand, physical demand, temporal demand, task complexity, situational stress, and distraction are specifically designed to capture workload in surgical settings. Figure 5 presents the SURG-TLX scores across these dimensions for the different experimental conditions.

All mean values for the SURG-TLX domains across conditions are summarized in Table 5 For each domain, the mean scores in the single-task conditions (st, st_d_) were lower than those in the multi-task conditions (mt, mt_d_, mt_BSS_, mt_direct_). Within the multi-task conditions, the highest domain-specific mean scores were observed in the mt and mt_d_ conditions, while comparatively lower mean scores were found in the mt_BSS_ and mt_direct_ conditions. This pattern was consistent across all six workload domains. The overall workload score, calculated as the average across all domains, showed the lowest mean in the st_d_ condition and the highest in the mt_d_ condition.

**Table 5:** Mean SURG-TLX scores (± standard deviation) across all domains and experimental conditions.

Condition/ domain	Mental demand	Physical demand	Temporal demand	Task complexity	Situational stress	Distraction	Overall
st	35.86 ± 20.70	29.31 ± 21.03	27.76 ± 19.89	27.76 ± 18.64	20.17 ± 20.51	25.17 ± 18.35	27.67 ± 20.15
st_d_	22.93 ± 19.25	25.34 ± 22.44	20.69 ± 19.58	26.72 ± 20.63	17.76 ± 20.64	18.97 ± 17.80	22.07 ± 20.08
mt	66.55 ± 17.73	41.72 ± 24.25	47.93 ± 18.88	64.31 ± 17.05	34.83 ± 23.24	56.38 ± 19.13	51.95 ± 23.05
mt_d_	67.07 ± 16.98	41.72 ± 24.10	53.97 ± 21.89	62.59 ± 21.03	42.24 ± 29.11	61.38 ± 21.17	54.83 ± 24.44
mt_BSS_	51.90 ± 23.96	35.00 ± 23.83	40.86 ± 24.20	53.10 ± 22.97	30.69 ± 25.10	44.83 ± 20.89	42.73 ± 24.60
mt_direct_	51.03 ± 22.65	38.62 ± 24.82	42.07 ± 21.82	53.97 ± 23.39	30.52 ± 22.41	33.45 ± 25.67	41.61 ± 24.70

The ‘Overall’ column represents the mean score across all six domains. Conditions include single-task (st, st_d_) and multi-task scenarios (mt, mt_d_, mt_BSS_, mt_direct_).

The two-way repeated measures ANOVA conducted to compare SURG-TLX scores across different task conditions and questionnaire domains revealed a significant main effect of condition, F(3.680, 103.027) = 38.957, *p* < .001, partial *η*^2^ = .582. A significant main effect was also found for the questionnaire domain, F(3.601, 100.830) = 17.838, *p* < .001, partial *η*^2^ = .389. *Post hoc* analyses indicated that the two single-task conditions (st and st_d_) did not differ significantly from each other in terms of SURG-TLX scores (*p* = .396), nor did the two multi-task conditions without sound enhancement algorithms (mt and mt_d_; *p* = 1.000), or the two multi-task conditions with sound enhancement algorithms (mt_BSS_ and mt_direct_; *p* = 1.000). However, both single-task conditions (st and st_d_) showed significantly lower SURG-TLX scores compared to the multi-task conditions, with all comparisons yielding *p*-values ≤ 0.007. Furthermore, a significant difference in SURG-TLX scores was observed between the multi-task conditions mt_d_ and mt_BSS_ (*p* = .002).

### 3.4 Comparison of subjective and objective data

**Figure 6:**
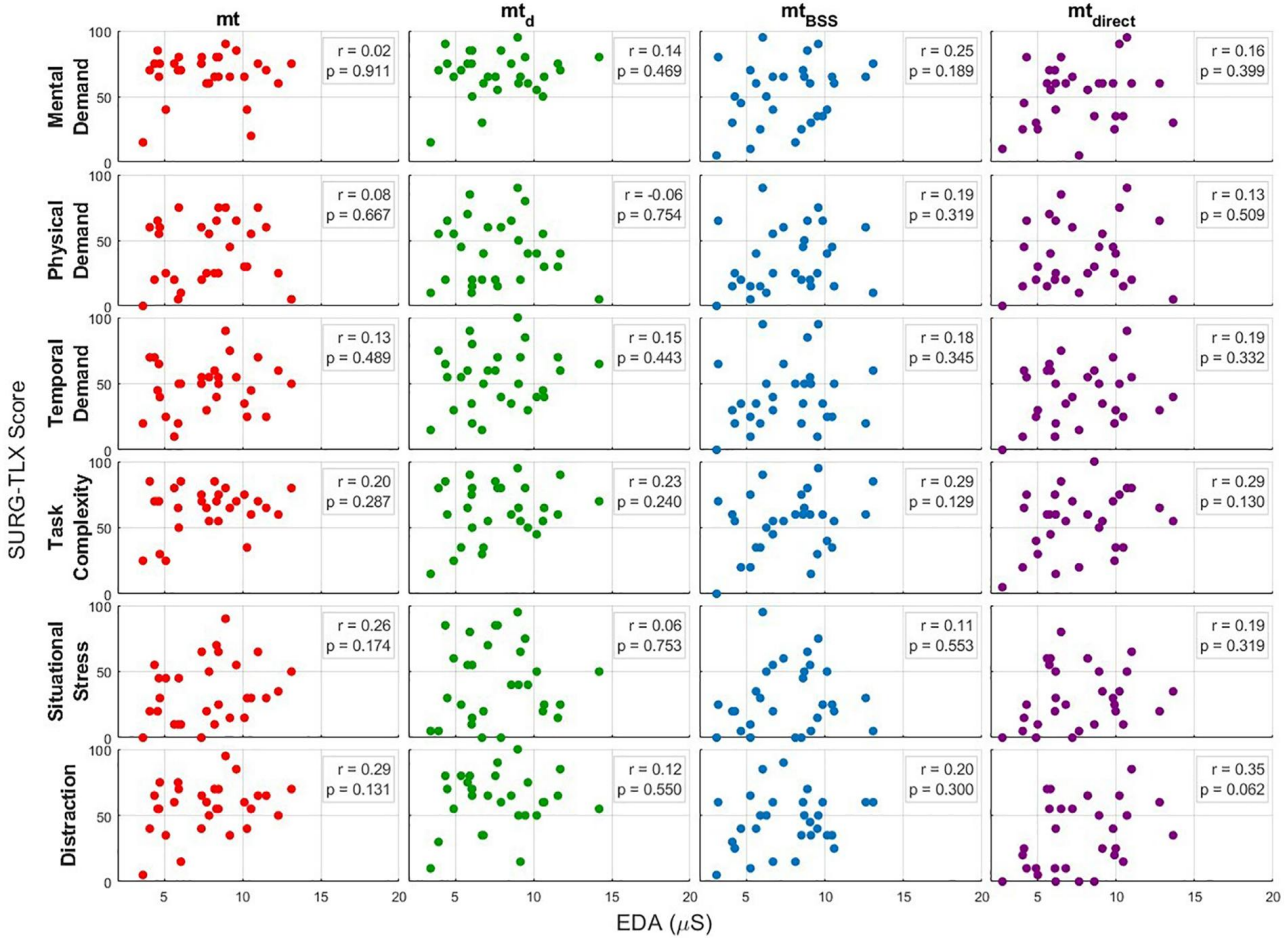
Correlation analysis between all domains of the SURG-TLX and measured EDA values across all multitasking conditions, r denotes the Pearson correlation coefficient, and *p* denotes the probability value for statistical significance. No correlations reached statistical significance, indicating limited correspondence between subjective workload and physiological stress measures.

To examine the relationship between physiological arousal and subjective workload, Pearson correlation analyses were conducted between EDA values and SURG-TLX ratings across six workload domains and four multitasking conditions (mt, mt_d_, mt_BSS_, and mt_direct_). Figure 6 illustrates the results of the correlation analyses between EDA and subjective workload ratings across six SURG-TLX domains and four multitasking conditions. Each subplot presents a scatterplot of EDA values plotted against SURG-TLX scores for a specific workload domain and condition, accompanied by the corresponding Pearson correlation coefficient (r) and *p*-value. The figure provides a visual summary of the strength and direction of associations between physiological arousal and perceived workload across different task and audio processing settings.

Overall, the results revealed consistently weak correlations between EDA and subjective workload scores. Correlation coefficients ranged from −0.061 to 0.351, with none reaching statistical significance (all *p*-values > 0.05). The strongest, though still non-significant, correlation was observed for the domain Distraction under the mt_direct_ condition (r = 0.351, *p* = 0.0621). Similarly, the domain Task Complexity showed moderate correlations under the mt_BSS_ and mt_direct_ conditions (r = 0.288, *p* = 0.1292 and r = 0.288, *p* = 0.1298), respectively. All remaining domains and conditions yielded weaker and statistically non-significant correlations (r < 0.26), indicating an overall absence of meaningful associations between EDA and perceived workload across the experimental settings.

## 4 Discussion

The present study aimed to compare EDA measurements at an alternative body site, specifically the shoulder and neck area, with the standard finger-based measurements during simulated surgical tasks. Heart rate and EDA were recorded to assess stress levels and evaluate the suitability of this alternative electrode placement. EDA data were compared between the two measurement sites. In addition, the impact of different noise reduction algorithms on stress and perceived workload was investigated to identify potential strategies for relieving strain during surgical procedures.

With regard to Aim (1), which focused on the validation of the shoulder-neck region as an alternative EDA measurement site, the results showed that the mean SCL measured at the shoulder and neck area were significantly lower than those recorded at the fingers. This finding is consistent with previous research reporting lower absolute EDA values at alternative sites due to reduced sweat gland density and activity (18, 20, 23). The fact that task conditions had no significant effect on SCL levels suggests either that SCL may not be sufficiently sensitive to short-term stress fluctuations in the context of this experiment, or that stress levels across the active conditions were relatively similar. Despite lower absolute values, consistent measurements at the shoulder demonstrate its potential as a reliable alternative site for EDA recording. Cross-correlation analysis showed a very high agreement between the shoulder and neck, with a correlation coefficient exceeding 0.99, indicating valid and reliable signal capture. These results support using the shoulder for EDA measurements, particularly when focusing on relative temporal changes or pattern detection. Although absolute values differ, the strong correlation confirms that stress-related variations in skin conductance are clearly reflected at the shoulder. This is particularly relevant for clinical settings, where palmar electrode placement is not feasible during surgical procedures. These findings are consistent with previous results obtained from a pilot study conducted in a similar setting (23). However, further validation under real surgical conditions remains an important next step.

With regard to Aim (2), which addressed the evaluation of smart hearing protection as a potential stress-reducing intervention, he significantly elevated heart rate observed across all active task conditions in comparison to rest confirms the influence of both cognitive and auditory load on physiological stress markers. This increase is in line with established findings in stress physiology (14, 16) and highlights the substantial stress that can arise even in simulated operating scenarios. Interestingly, no significant differences were found between the individual active conditions. While subjective workload measures clearly differentiated between conditions, EDA and heart rate showed limited sensitivity to these differences. Therefore, the current findings should be interpreted primarily as demonstrating the feasibility and construct validity of the measurement and intervention framework, rather than as providing strong evidence for physiological efficacy of the smart hearing protection. This suggests that each scenario produced a similar level of physiological activation, possibly reflecting a general baseline workload in surgical settings or indicating that the experimental design, particularly the multitasking tasks, consistently elicited a high level of stress. The SURG-TLX results clearly indicate that perceived workload was significantly higher under multitasking conditions than during single-task conditions. Participants were particularly responsive to increased cognitive demands, situational stress, and distraction. These findings confirm the sensitivity of the SURG-TLX in detecting subjective workload in surgical contexts (8). Of particular note is that the use of sound enhancement technologies, such as mt_BSS_ and mt_direct_, was associated with a reduction in perceived workload. This suggests that technical tools, including noise reduction and speech enhancement algorithms, may contribute to improvements in the surgical work environment by reducing acoustic distractions and enhancing focus. The significant difference between the mt_d_ and mt_BSS_ conditions further supports the effectiveness of selective noise suppression. These improvements are particularly important considering the potential impact of stress on patient safety and surgeon health (2, 4).

The lack of significant correlations between objective physiological stress indicators such as EDA and subjective stress ratings obtained from the SURG-TLX is not uncommon. It reflects the complex nature of stress perception. Physiological parameters typically capture immediate and automatic stress responses, whereas subjective assessments are influenced by personal appraisal, coping strategies, and contextual factors (16, 17). Furthermore, it should be considered that subjective assessments may be influenced by expectancy effects and response bias (29). Participants might tend to provide answers they believe are expected—for example, that multitasking conditions should be perceived as particularly stressful. Similar findings have been reported in other studies across different fields and with varying stress measurement methods, showing no clear correlation between subjective and objective stress markers (7, 30, 31). The weak and in some cases only trend-level associations found in this study, particularly regarding distraction and task complexity, suggest that future research should consider more specific measurement approaches or longer observation periods to better capture these dynamics.

A major limitation of the present study is that the experimental environment was designed as a simulation. This may restrict the generalizability of the findings to real surgical settings, where complex, dynamic, and partly unpredictable stressors occur that cannot be fully replicated (1). In an actual operating room, additional factors, such as time pressure, interactions within the surgical team, or patient-specific risks, act alongside the cognitive and motor demands of the task and can significantly influence both stress levels and workload (32). Furthermore, the physiological responses of participants under real-world conditions may differ from those observed in the laboratory. In addition, the study was not conducted with medical professionals. This decision was made deliberately in order to avoid placing demands on limited personnel resources and to first establish an appropriate measurement methodology under controlled laboratory conditions. Consequently, the findings should be interpreted primarily as methodological validation and proof-of-concept rather than as evidence of direct clinical effectiveness. Nevertheless, it is reasonable to assume that experienced surgical staff, due to their routine, professional expertise, and specific exposure to workload, might respond differently to the experimental conditions compared to laypersons. It should also be noted that EDA, although widely used in numerous studies as a reliable indicator of psychophysiological stress, cannot unequivocally determine the precise cause of stress in each individual case (18). Beyond the experimentally induced workload, other factors, such as individual daily condition, emotional state, or nonspecific physiological reactions, may also affect the measured stress level (33). In addition, it should be noted that a large proportion of the study participants were female, and the potential influence of hormonal fluctuations across the menstrual cycle on physiological stress markers was not controlled for. Previous research has shown that hormonal variations, particularly fluctuations in estrogen and progesterone levels, can affect autonomic nervous system activity and electrodermal responses (34). As the current study did not assess or control for menstrual cycle phase or hormonal status, this may have introduced additional variability in the physiological data, particularly in EDA and heart rate measurements. Consequently, this factor should be considered as a potential limitation when interpreting the physiological results.

Nonetheless, the controlled simulation setting offered the advantage of minimizing confounding variables and ensuring standardized comparability between the investigated conditions. This provided a solid foundation for validating the applied measurement methods and exploring initial approaches for potential intervention strategies. To enhance the validity and applicability of the results, future research should not only include larger and more diverse samples but also incorporate real clinical environments and medical professionals.

In summary, EDA measurement at the shoulder and neck area represents a promising and practical alternative to palmar measurement for surgical contexts. The use of intelligent sound enhancement algorithms has the potential to reduce subjective workload, improve working conditions, and promote well-being among surgical personnel. Future studies should aim to validate these findings in real operating room environments and investigate the long-term effects on stress and health outcomes.

## 5 Conclusion

This study demonstrates that subjective workload during surgical tasks is significantly influenced by acoustic conditions in the operating room. While smart hearing protection reduced both subjective workload and electrodermal activity, the absence of a strong correlation between physiological and subjective measures emphasizes the need for a combined assessment approach. Future research should explore long-term effects and investigate the integration of such stress-reducing technologies into clinical routine.

## Data Availability

The raw data supporting the conclusions of this article will be made available by the authors, without undue reservation.
